# Supplementary material for: Vanadium(IV) Complexes with Methyl-Substituted 8-Hydroxyquinolines: Catalytic Potential in the Oxidation of Hydrocarbons and Alcohols with Peroxides and Biological Activity
Source: Molecules. 2021 Oct 21;26(21):6364. doi: 10.3390/molecules26216364 (PMC8588223; doi:10.3390/molecules26216364)
Supplement: Supplementary file 1 [file molecules-26-06364-s001.zip › molecules-1414482-supplementary.pdf]

Electronic Supplementary Information

Article

Vanadium Complexes with Methyl-substituted 8-Hydroxyquinolines: Catalytic Potential in the Oxidation of Hydrocarbons and Alcohols with Peroxides and Biological Activity

Joanna Palion-Gazda[1], André Luz[2,3], Luis R. Raposo[2,3], Katarzyna Choroba[1], Jacek E. Nycz[1], Alina Bieńko[4], Agnieszka Lewińska[4], Karol Erfurt[5], Pedro V. Baptista[2,3], Barbara Machura[1,*], Alexandra R. Fernandes[2,3*], Lidia S. Shul'pina[6], Nikolay S. Ikonnikov[6], Georgiy B. Shul'pin [7,8,*]

[1]Institute of Chemistry, University of Silesia, Szkolna 9, 40-006 Katowice, Poland. Email: barbara.machura@us.edu.pl
[2]Associate Laboratory i4HB - Institute for Health and Bioeconomy, NOVA School of Science and Technology, NOVA University Lisbon, 2819-516 Caparica, Portugal. Email: ma.fernandes@fct.unl.pt
[3]UCIBIO – Applied Molecular Biosciences Unit, Department of Life Sciences, NOVA School of Science and Technology, NOVA University Lisbon, 2819-516 Caparica, Portugal
[4]Faculty of Chemistry, University of Wroclaw, F. Joliot-Curie 14, 50-383 Wroclaw, Poland
[5]Department of Chemical Organic Technology and Petrochemistry, Silesian University of Technology, Krzywoustego 4, 44-100 Gliwice, Poland
[6]A.N. Nesmeyanov Institute of Organoelement Compounds, Russian Academy of Sciences, ulitsa Vavilova 28, Moscow 119991, Russia, e-mail: shulpina@ineos.ac.ru
[7]N.N .Semenov Federal Research Center for Chemical Physics, Russian Academy of Sciences, ulitsa Kosygina 4, Moscow 119991, Russia ,e-mail: gbsh@mail.ru
[8]Chair of Chemistry and Physics, Plekhanov Russian University of Economics, Stremyannyi pereulok 36, Moscow 117997, Russia
*Correspondence: barbara.machura@us.edu.pl, ma.fernandes@fct.unl.pt; gbsh@mail.ru

Table of Contents

General Characterization	
IR spectra of complexes **1**–**3** and free ligands	Figure S1
HRMS of complexes **1**–**3**	Figure S2
X-ray studies	
Selected bond lengths and angles for **1**–**3**	Table S1-S3
Short intra- and intermolecular hydrogen bonds.	Table S4
Short π•••π interactions.	Table S5
X—Y•••Cg(J)(π-ring) interactions.	Table S6
Hirshfeld surface and 2D fingerprint plot for **1**–**3**, together with the relative contributions of various intermolecular interactions to the Hirshfeld surface	Figure S3-S5
View of the intermolecular interactions and packing in **1**–**3**.	Figure S6-S8
The selected structural parameters of five-coordinated vanadium(IV) complexes	Table S7
X–ray powder diffraction patterns of **3**	Figure S9
EPR spectra	
EPR frozen solution spectra (at 77 K) of compounds 1–3; in acetonitrile	Figure S10
Absorption spectroscopy	
UV-Vis spectra of complexes **1**–**3**	Figure S11
UV-Vis stability spectra in DMSO of **1**–**3**	Figure S12
Catalytic data	
Oxidation of cyclohexane catalyzed by oxido-V/PCA/aq.H2O2/CH3CN systems	Table S8
Biological results	
Antiproliferative effect of complexes 1-3 in normal dermal fibroblasts, after 48 h. evaluated by the MTS method.	Figure S13
Cell viability of HCT116 cells.	Figure S14

General Characterization

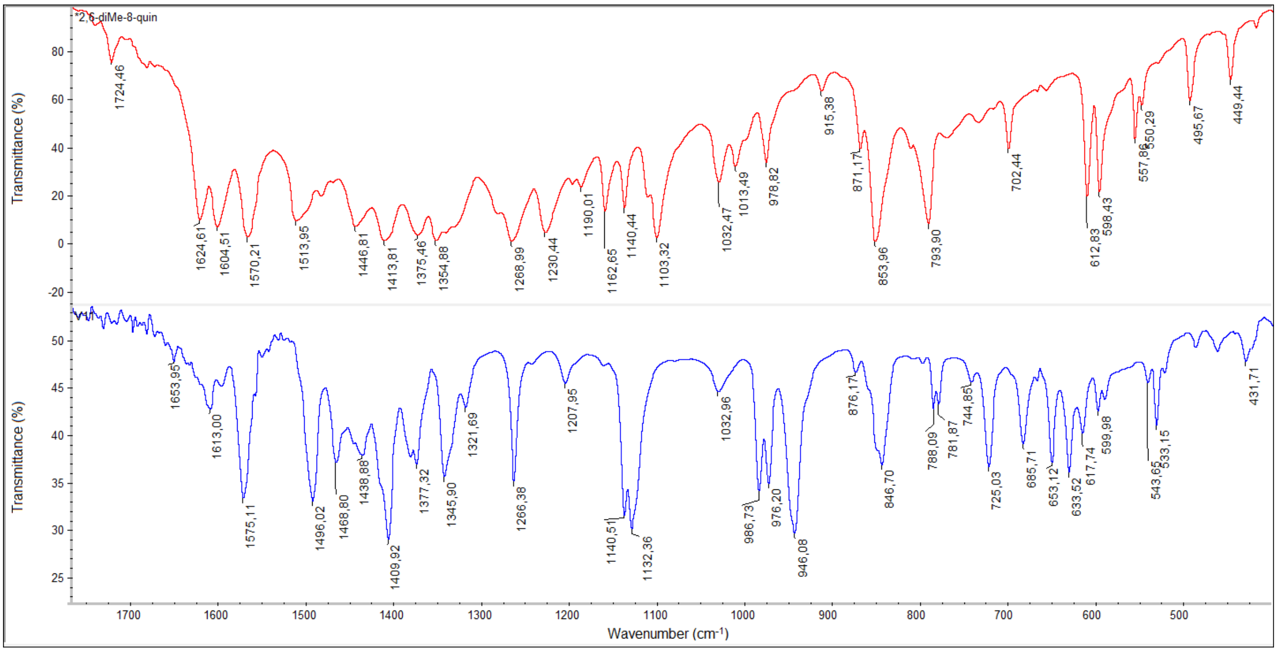

(a)

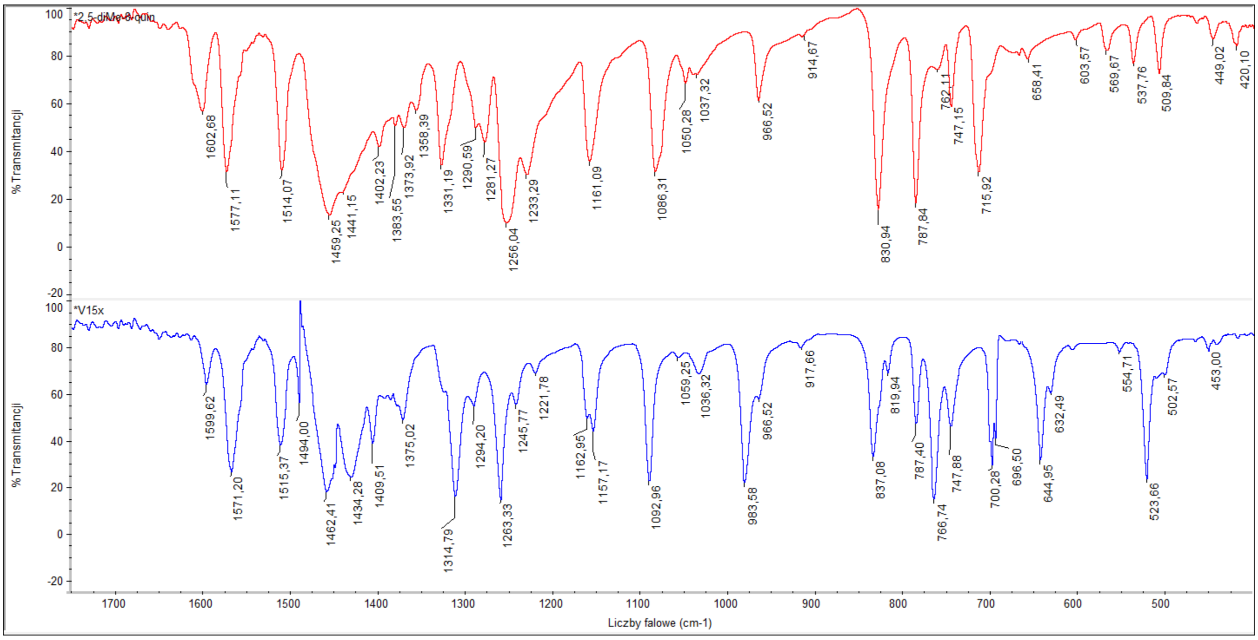

(b)

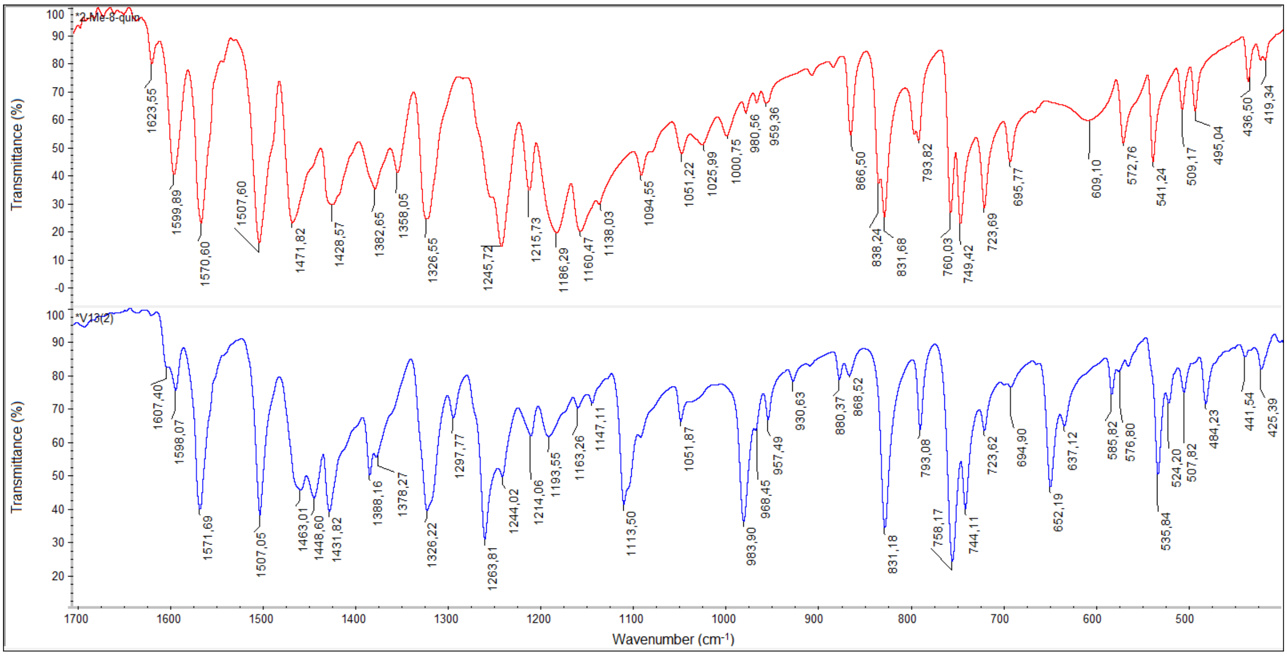

(c)

Figure S1. IR spectra of the complexes **1–3** (blue) and free ligands (red) (a-c).

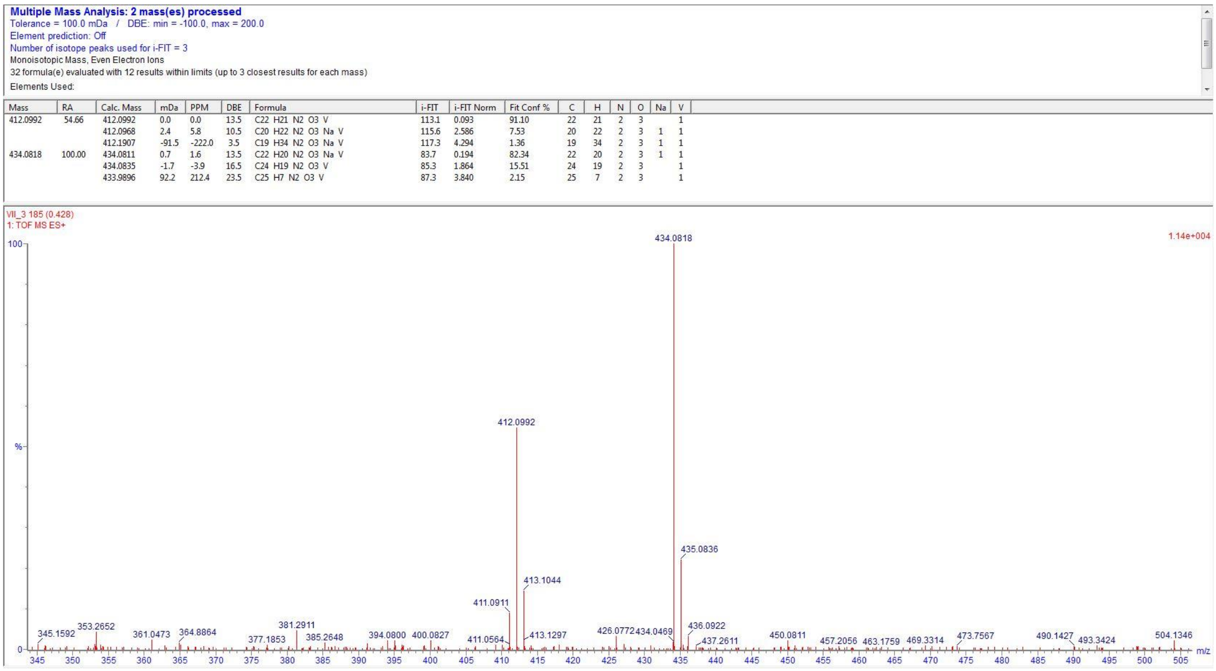

Multiple Mass Analysis: 2 mass(es) processed
Tolerance = 100.0 mDa / DBE: min = -100.0, max = 200.0
Element prediction: Off
Number of isotope peaks used for i-FIT = 3
Monoisotopic Mass, Even Electron Ions
32 formula(e) evaluated with 12 results within limits (up to 3 closest results for each mass)
Elements Used:

Mass	RA	Calc. Mass	mDa	PPM	DBE	Formula	i-FIT	i-FIT Norm	Fit Conf %	C	H	N	O	Na	V
412.0992	54.66	412.0992	0.0	0.0	13.5	C22 H21 N2 O3 V	113.1	0.093	91.10	22	21	2	3		1
		412.0968	2.4	5.8	10.5	C20 H22 N2 O3 Na V	115.6	2.586	7.53	20	22	2	3	1	1
		412.1907	-91.5	-222.0	3.5	C19 H34 N2 O3 Na V	117.3	4.294	1.36	19	34	2	3	1	1
434.0818	100.00	434.0811	0.7	1.6	13.5	C22 H20 N2 O3 Na V	83.7	0.194	82.34	22	20	2	3	1	1
		434.0835	-1.7	-3.9	16.5	C24 H19 N2 O3 V	85.3	1.864	15.51	24	19	2	3		1
		433.9896	92.2	212.4	23.5	C25 H7 N2 O3 V	87.3	3.840	2.15	25	7	2	3		1

(a)

HRMS (ESI): calcd for $C_{22}H_{20}N_2O_3NaV^+$ $[M+Na]^+$ 434.0811 found 434.0818.

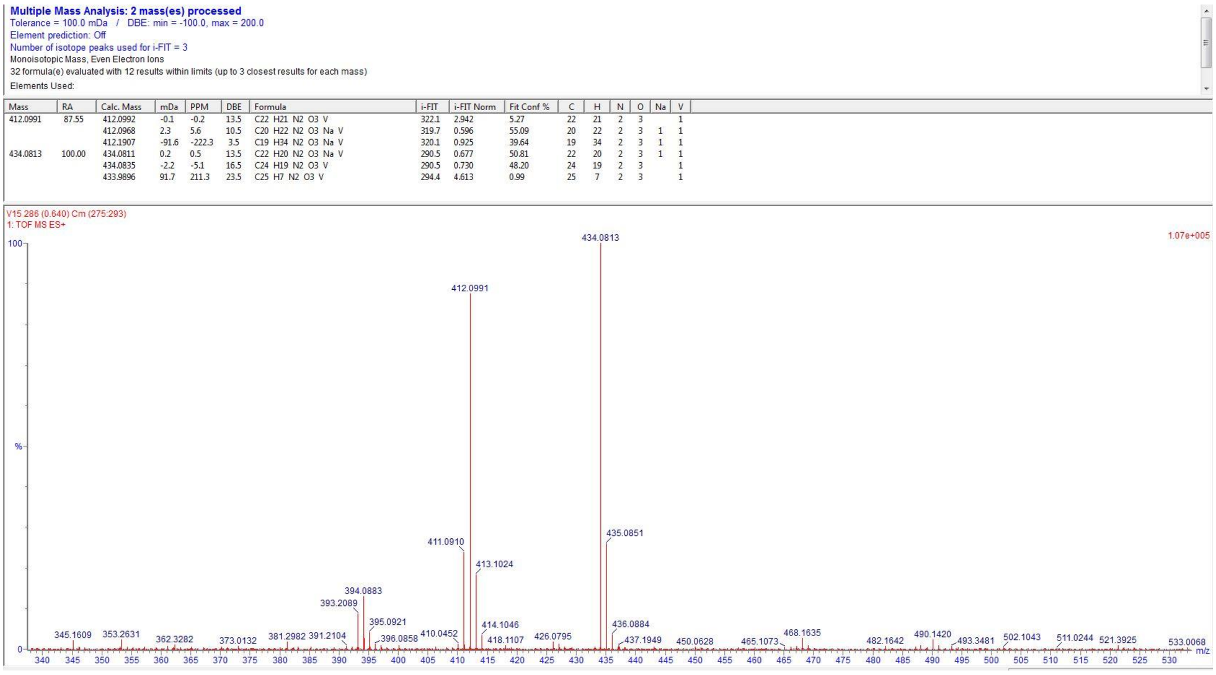

Multiple Mass Analysis: 2 mass(es) processed
Tolerance = 100.0 mDa / DBE: min = -100.0, max = 200.0
Element prediction: Off
Number of isotope peaks used for i-FIT = 3
Monoisotopic Mass, Even Electron Ions
32 formula(e) evaluated with 12 results within limits (up to 3 closest results for each mass)
Elements Used:

Mass	RA	Calc. Mass	mDa	PPM	DBE	Formula	i-FIT	i-FIT Norm	Fit Conf %	C	H	N	O	Na	V
412.0991	87.55	412.0992	-0.1	-0.2	13.5	C22 H21 N2 O3 V	322.1	2.942	5.27	22	21	2	3		1
		412.0968	2.3	5.6	10.5	C20 H22 N2 O3 Na V	319.7	0.596	55.09	20	22	2	3	1	1
		412.1907	-91.6	-222.3	3.5	C19 H34 N2 O3 Na V	320.1	0.925	39.64	19	34	2	3	1	1
434.0813	100.00	434.0811	0.2	0.5	13.5	C22 H20 N2 O3 Na V	290.5	0.677	50.81	22	20	2	3	1	1
		434.0835	-2.2	-5.1	16.5	C24 H19 N2 O3 V	290.5	0.730	48.20	24	19	2	3		1
		433.9896	91.7	211.3	23.5	C25 H7 N2 O3 V	294.4	4.613	0.99	25	7	2	3		1

(b)

HRMS (ESI): calcd for $C_{22}H_{20}N_2O_3NaV^+$ $[M+Na]^+$ 434.0811 found 434.0813.

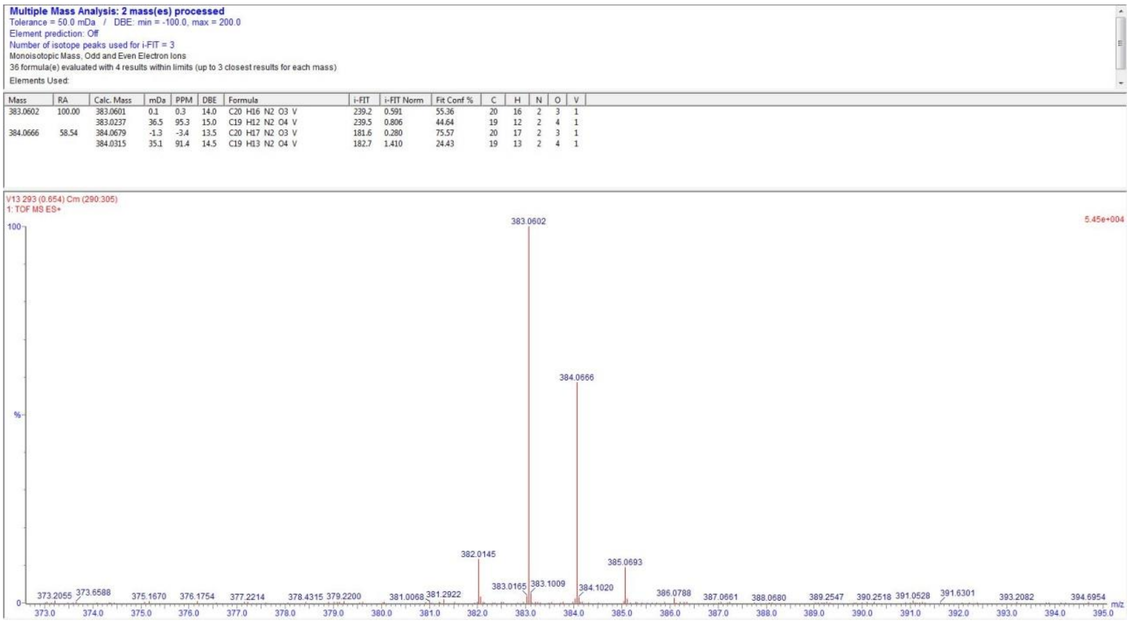

Multiple Mass Analysis: 2 mass(es) processed
Tolerance = 50.0 mDa / DBE: min = -100.0, max = 200.0
Element prediction: Off
Number of isotope peaks used for i-FIT = 3
Monoisotopic Mass, Odd and Even Electron Ions
36 formula(e) evaluated with 4 results within limits (up to 3 closest results for each mass)
Elements Used:

Mass	RA	Calc. Mass	mDa	PPM	DBE	Formula	i-FIT	i-FIT Norm	Fit Conf %	C	H	N	O	V
383.0602	100.00	383.0601	0.1	0.3	14.0	C20 H16 N2 O3 V	239.2	0.591	55.36	20	16	2	3	1
		383.0237	36.5	95.3	15.0	C19 H12 N2 O4 V	239.5	0.806	44.64	19	12	2	4	1
384.0666	58.54	384.0679	-1.3	-3.4	13.5	C20 H17 N2 O3 V	181.6	0.280	75.57	20	17	2	3	1
		384.0315	35.1	91.4	14.5	C19 H13 N2 O4 V	182.7	1.410	24.43	19	13	2	4	1

(c)

HRMS (ESI): calcd for $C_{20}H_{16}N_2O_3V$ $[M]^+$ 383.0601 found 383.0602.

Figure S2. HRMS spectra of the complexes **1–3** (a-c)

X-Ray studies

Table S1. Selected bond lengths (Å) and angles (deg) for **1**.

Bond lengths		Bond angles	
V(1)–O(1)	1.9210(14)	O(1)a–V(1)–O(1)	125.26(9)
V(1)–O(2)	1.590(2)	O(1)–V(1)–O(2)	117.37(5)
V(1)–N(1)	2.1239(15)	O(1)–V(1)–N(1)	80.51(6)
		O(1)a–V(1)–N(1)	89.93(6)
		O(2)–V(1)–N(1)	100.40(4)
		N(1)–V(1)–N(1)a	159.19(9)

[a]Symmetry transformations used to generate equivalent atoms: (a) = *-x,y,1/2-z*

Table S2. Selected bond lengths (Å) and angles (deg) for **2**.

Bond lengths		Bond angles	
V(1)–O(1)	1.924(2)	O(1)–V(1)–O(2)	126.96(11)
V(1)–O(2)	1.917(2)	O(1)–V(1)–O(3)	116.84(11)
V(1)–O(3)	1.595(3)	O(2)–V(1)–O(3)	116.20(12)
V(1)–N(1)	2.122(2)	O(1)–V(1)–N(1)	80.07(9)
V(1)–N(2)	2.125(2)	O(2)–V(1)–N(1)	91.91(9)
		O(3)–V(1)–N(1)	98.56(11)
		O(1)-V(1)-N(2)	90.18(9)
		O(2)-V(1)-N(2)	80.51(10)
		O(3)–V(1)–N(2)	100.85(11)
		N(1)–V(1)–N(2)	160.56(11)

Table S3. Selected bond lengths (Å) and angles (deg) for **3**.

Bond lengths		Bond angles	
V(1)–O(1)	1.9139(13)	O(1)[a]–V(1)–O(1)	127.03(9)
V(1)–O(2)	1.588(2)	O(1)–V(1)–O(2)	116.48(5)
V(1)–N(1)	2.1308(16)	O(1)–V(1)–N(1)	80.31(6)
		O(1)[a]–V(1)–N(1)	91.33(6)
		O(2)–V(1)–N(1)	99.36(4)
		N(1)–V(1)–N(1)[a]	161.29(9)

Table S4. Short intra- and intermolecular hydrogen bonds.

D–H•••A	D–H [Å]	H•••A [Å]	D–A [Å]	D–H•••A [°]
**1**				
C(10)–H(10A)•••O(1)[a]	0.96	2.56	3.237(3)	128.0
C(2)–H(2)•••O(2)[b]	0.93	2.66	3.142(3)	112.9
**2**				
C(10)–H(10C)•••O(2)	0.96	2.50	3.084(4)	119.0
C(21)–H(21C)•••O(1)	0.96	2.36	3.185(5)	144.0
C(22)–H(22A)•••O(3)[c]	0.96	2.49	3.399(5)	158.0
**3**				
C(10)–H(10C)•••O(1)	0.96	2.33	3.503(15)	145.2

Symmetry codes: (a) = *–x,y*,1/2–*z;* (b) = *–x*,1-*y,–z;* (c) = 2–*x*,1–*y*,2–*z;* (d) = 1–x,y,1/2–z

Table S5. Short π•••π interactions.

Cg(I)•••Cg(J)	Cg(I)•••Cg(J) [Å]	α [°]	β [°]	γ [°]	Cg(I)-Perp [Å]	Cg(J)-Perp [Å]
**1**						
Cg(1)•••Cg(1)[a]	3.5193(12)	0	12.33	12.33	3.4381(8)	3.4381(8)
Cg(1)•••Cg(2)[a]	3.9845(13)	0.42(10)	30.38	30.36	3.4382(8)	3.4375(9)
**2**						
Cg(1)•••Cg(2)[b]	3.8928(18)	0.94(15)	29.2	29.1	3.4024(13)	3.3991(13)
Cg(3)•••Cg(3)[c]	3.8328(19)	0.00(15)	6.4	6.4	3.8091(13)	3.8090(13)
Cg(2)•••Cg(2)[b]	3.7055(19)	0.02(15)	23.9	23.9	3.3885(13)	3.3885(13)
**3**						
Cg(4)•••Cg(4)[b]	3.6285(12)	0	10.77	10.77	3.5645(8)	3.5645(8)

α = dihedral angle between *Cg*(I) and *Cg*(J); *Cg*(I)-Perp = Perpendicular distance of *Cg*(I) on ring J; *Cg*(J)-Perp = perpendicular distance of *Cg*(J) on ring I; β = angle *Cg*(I)→*Cg*(J) vector and normal to ring I; γ = angle *Cg*(I) →*Cg*(J) vector and normal to plane J;

Cg1 is the centroid of atoms N(1)/C(1)/C(2)/C(3)/C(4)/C(9); Cg2 is the centroid of atoms C(4)/C(5)/C(6)/C(7)/C(8)/C(9); Cg3 is the centroid of atoms N(2)/C(12)/C(13)/C(14)/C(15)/C(16); Cg4 is the centroid of atoms N(1)/C(1)/C(2)/C(3)/C(4)/C(5);

Symmetry codes: (a) = -*x*,2-*y*,-*z*; (b) = 1-*x*,1-*y*,1-*z*; (c) = 2-*x*,1-*y*,2-*z*

Table S6. X—Y•••Cg(J)(π-ring) interactions.

Y-X(I)•••Cg(J)	X(I)•••Cg(J) [Å]	X-Perp [Å]	γ [°]	Y-X(I)•••Cg(J) [°]
1				
C(10)–H(10C)•••Cg(1)a	2.91	-2.86	9.70	140.0
2				
C(10)–H(10B)•••Cg(3)b	2.98	-2.89	14.41	136.0
3				
C(10)–H(10A)•••Cg(3)c	2.78	-2.73	11.42	165.0

γ = angle X(I)→Cg(J) vector and normal to plane J.

Cg1 is the centroid of atoms N(1)/C(1)/C(2)/C(3)/C(4)/C(9); Cg3 is the centroid of atoms N(1)/C(1)/C(2)/C(3)/C(4)/C(5);

Symmetry codes: (a) = *-x,1-y,-z;* (b) = 1-*x*,1/2+*y*,3/2-*z;* (c) = 1-*x*,–*y*,1-*z*;

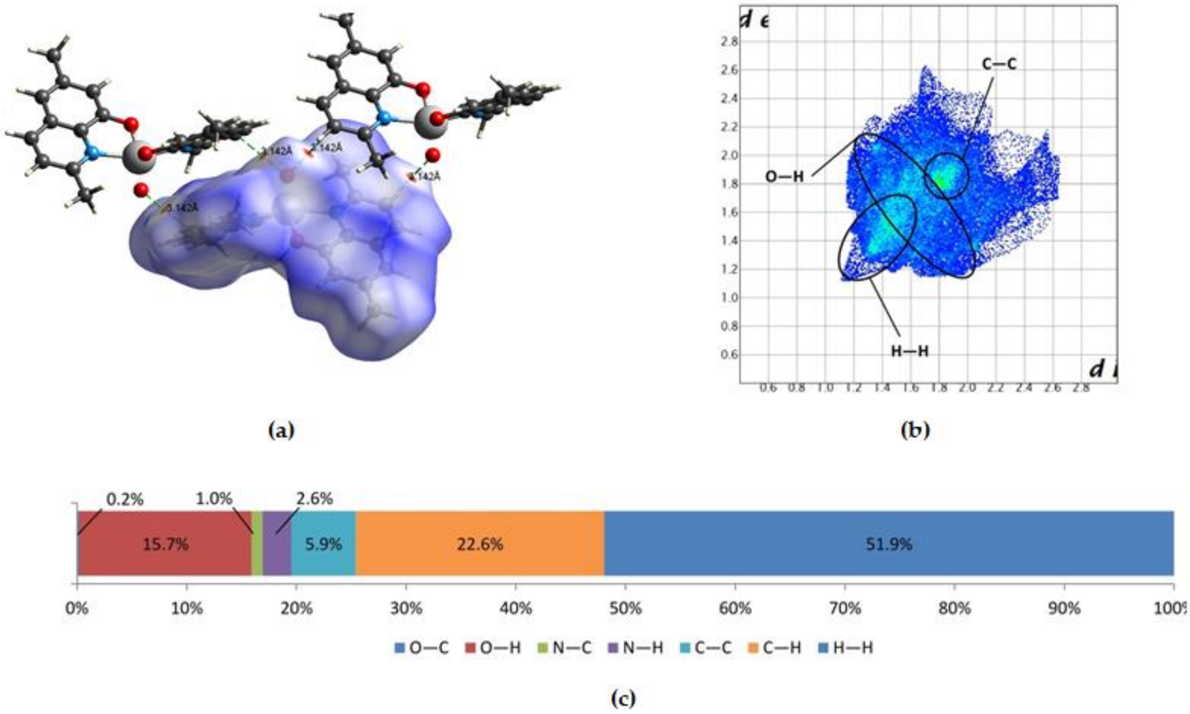

Figure S3. Hirshfeld surface mapped with d_{norm} along with intermolecular hydrogen bonds (a), and 2D fingerprint plot (b) for **1**, together with the relative contributions of various intermolecular interactions to the Hirshfeld surface (c).

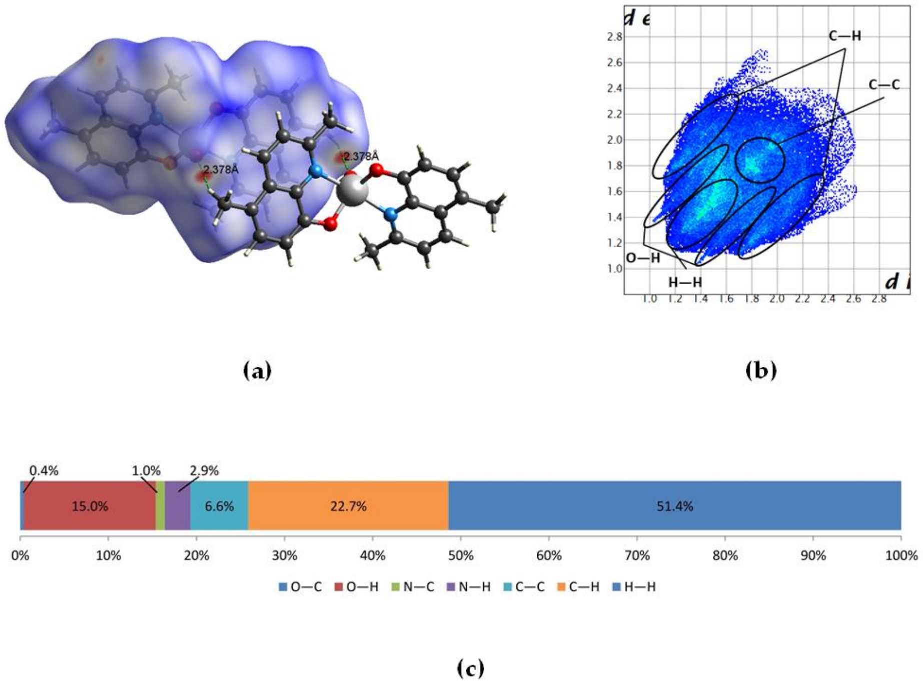

Figure S4. Hirshfeld surface mapped with d_{norm} along with intermolecular hydrogen bonds (a), and 2D fingerprint plot (b) for **2**, together with the relative contributions of various intermolecular interactions to the Hirshfeld surface (c).

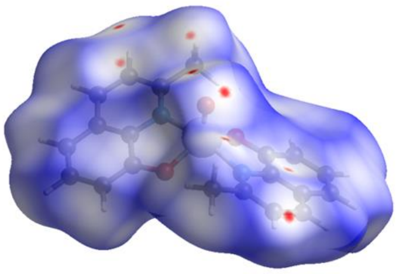

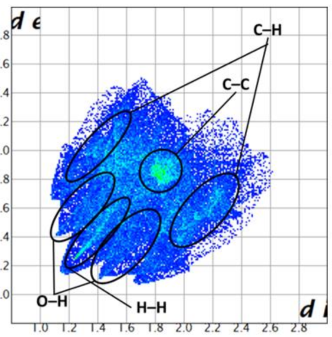

(a) **(b)**

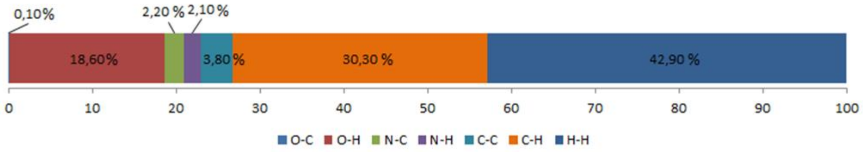

(c)

Figure S5. Hirshfeld surface mapped with dnorm along with intermolecular hydrogen bonds (a), and 2D fingerprint plot (b) for 3, together with the relative contributions of various intermolecular interactions to the Hirshfeld surface (c).

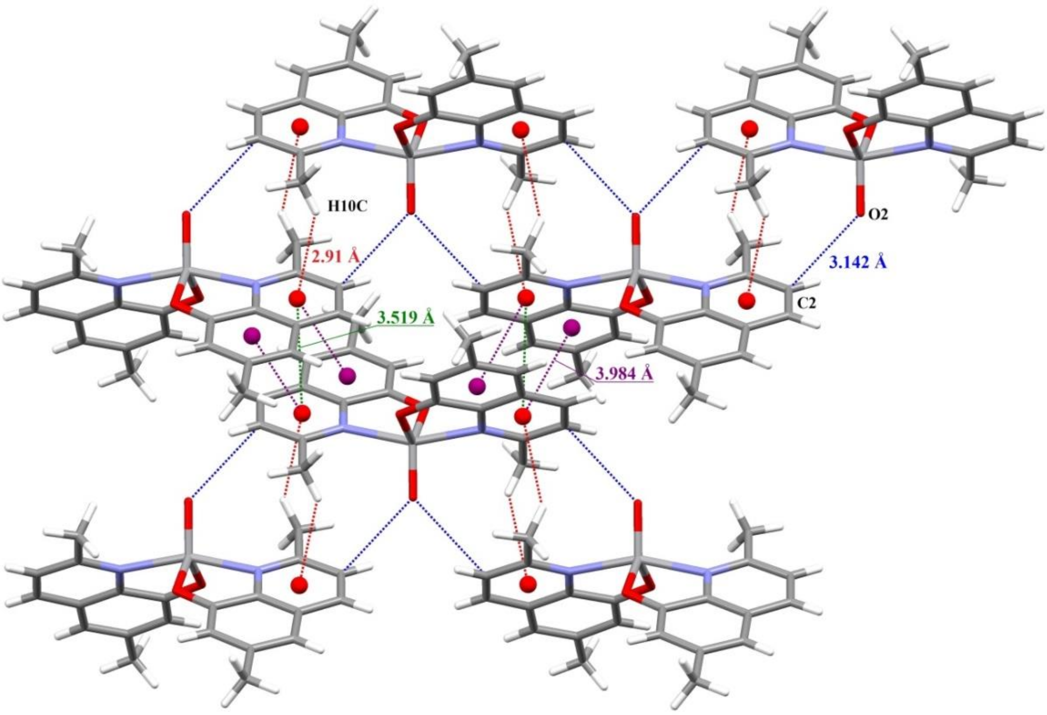

(a)

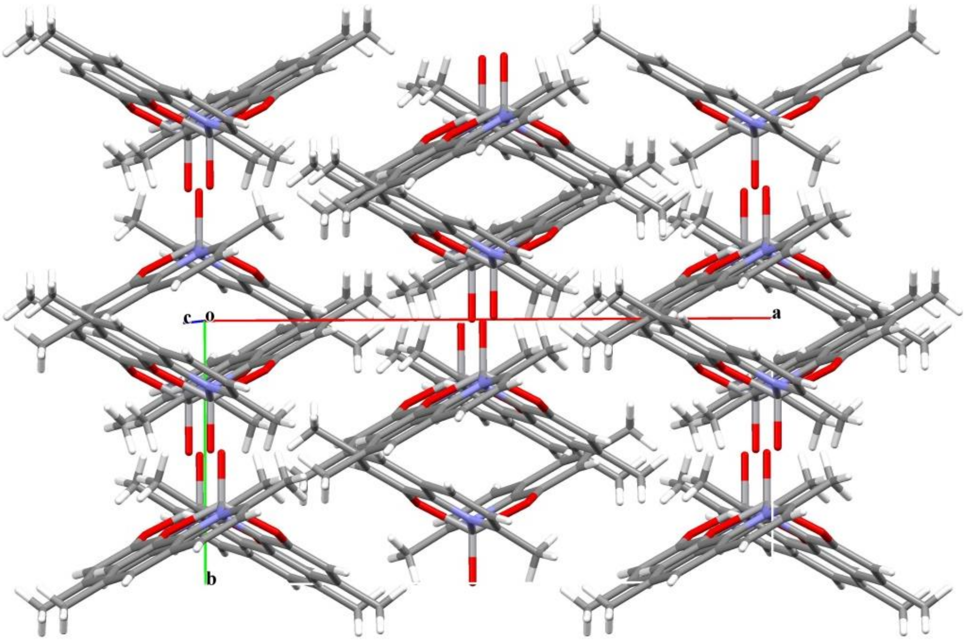

(b)

Figure S6. View of the intermolecular interactions (π•••π, C–H•••π and C–H•••O) in 1 (a), view of the supramolecular packing of 1 alongside c (b).

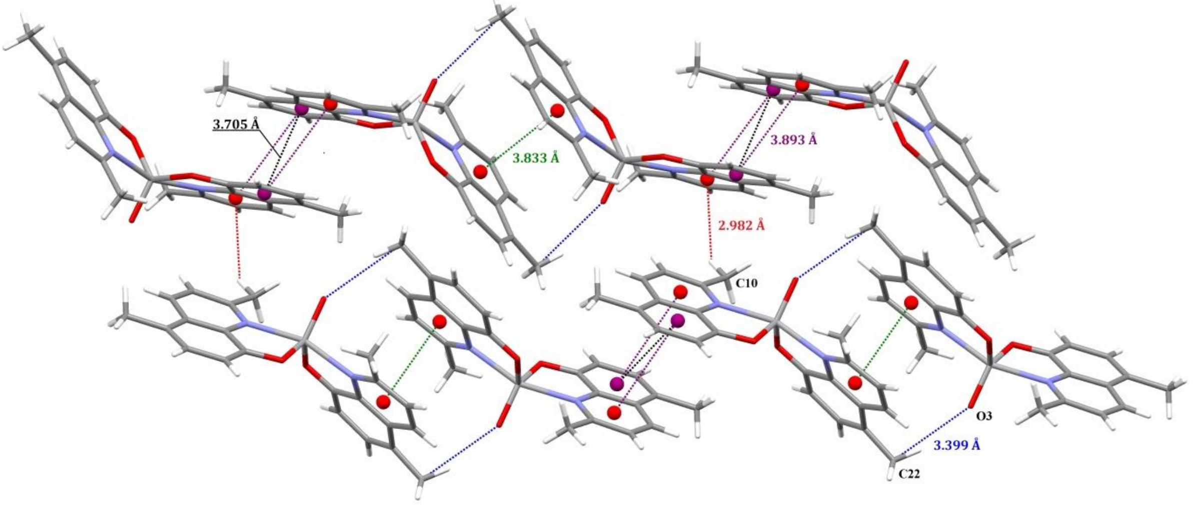

(a)

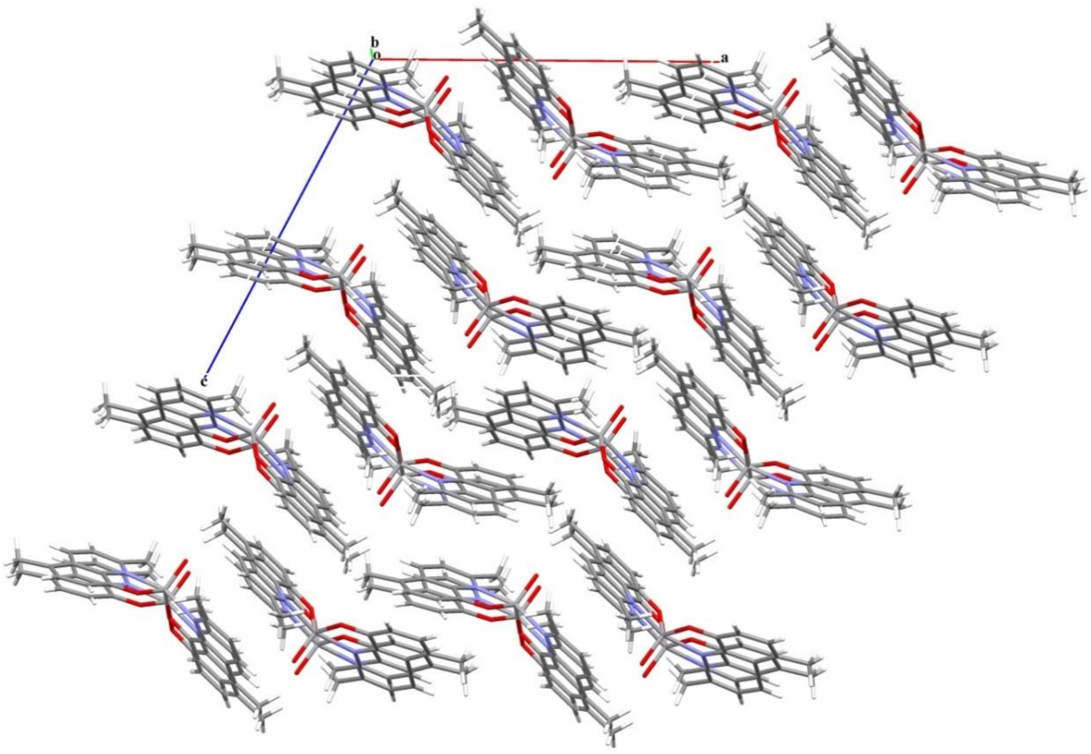

(b)

Figure S7. View of the intermolecular interactions (π•••π, C–H•••π and C–H•••O) in 2 (a), view of the supramolecular packing of 2 alongside *b* (b).

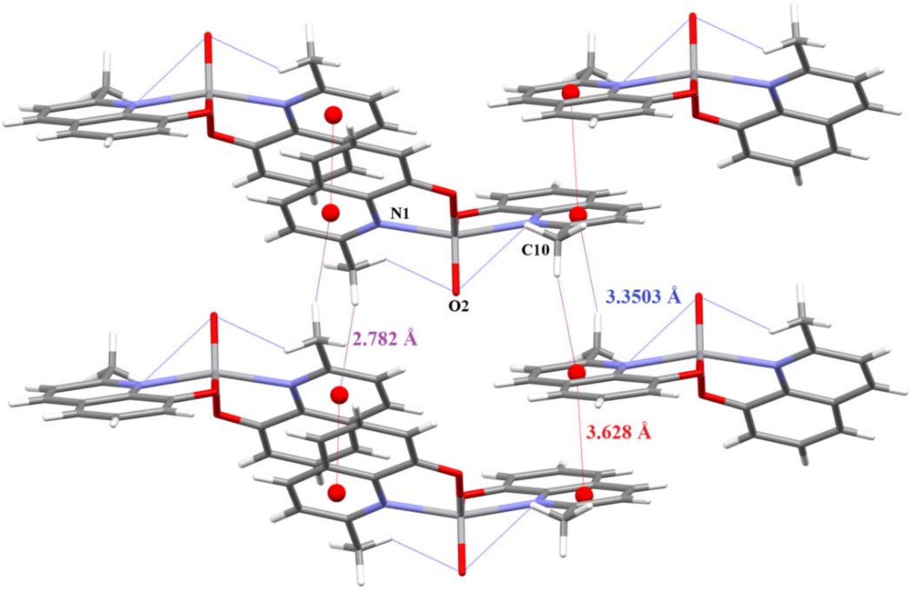

(a)

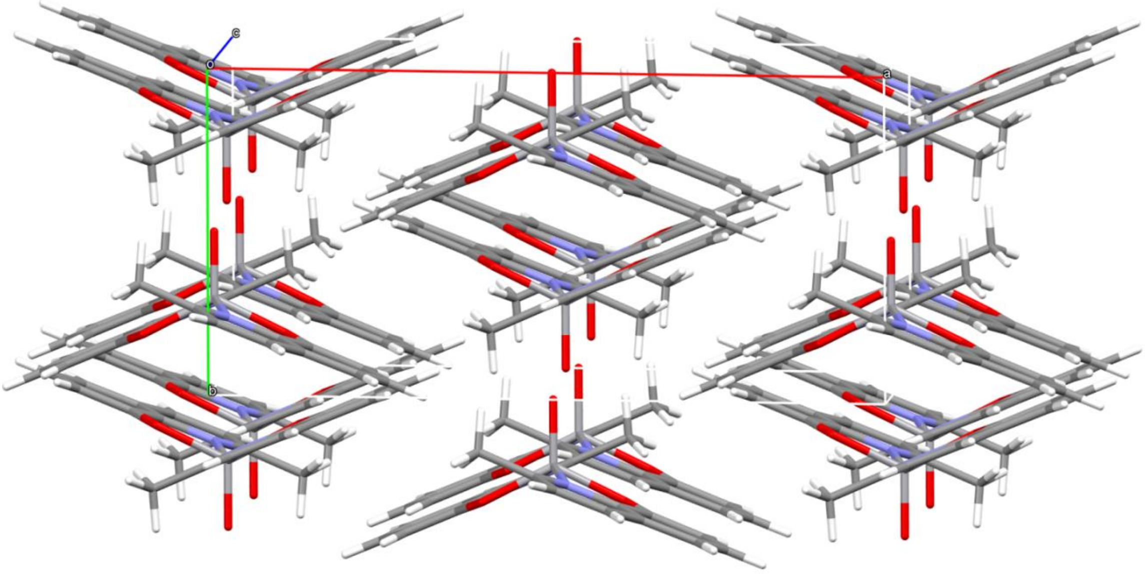

(b)

Figure S8. View of the intermolecular interactions (π•••π, C–H•••π and C–H•••O) in **3** (a), view of the supramolecular packing of **3** alongside *c* (b).

Table S7. The selected structural parameters of five-coordinated vanadium(IV) complexes [VO(N⌒O)$_2$] and [VO(O⌒N⌒N⌒O)].

Compound	V=O [Å]	V–O [Å]	V–N [Å]	τ	Ref.
[VO(N⌒O)$_2$]					
[VO(2,6-(Me)$_2$-quin)$_2$] (1)	1.590(2)	1.9210(14)	2.1239(15)	0.57	this work
[VO(2,5-(Me)$_2$-quin)$_2$] (2)	1.595(3)	1.924(2); 1.917(2)	2.122(2); 2.125(2)	0.56	this work
[VO(2-Me-quin)$_2$)]	1.600(8)	1.921(5)	2.136(6)	0.56	1
[VO(2-Me-5-SMe-quin)$_2$)]	1.576(6)	1.934(6); 1.923(6)	2.106; 2.122(8)	0.50	2
[VO(n-Pr-sal)$_2$]	1.5945(18)	1.9042(16); 1.8976(19)	2.126(2); 2.107(2)	0.53	3
[VO(L^1)$_2$]	1.587(4)	1.901 (4); 1.914 (3)	2.087 (4); 2.088 (4)	0.58	4
[VO(2-OC$_6$H$_4$CHNH)$_2$]	1.589(4)	1.917 (4); 1.919(4)	2.056(6); 2.058(6)	0.22	5
[VO(L^2)$_2$]	1.5987(16)	1.9038(16); 1.9194(17)	2.1088(19); 2.1095(18)	0.37	6
[VO(L^3)$_2$]	1.589 (2)	1.891(2); 1.904(2)	2.110(2); 2.121(2)	0.42	7
[VO{(S)-Clsal-am}$_2$]·C$_7$H$_8$	1.592(5)	1.912(4); 1.915(4)	2.076(6); 2.085(6)	0.62	
[VO{(S)-naph-am}$_2$]	1.611(2)	1.900(3); 1.904(3)	2.065(3); 2.085(3)	0.75	8
[VO{(R)-sal-am}$_2$]·C$_7$H$_8$	1.592(2)	1.905(2); 1.910(2)	2.112(3); 2.100(3)	0.44	
[VO(L^4)$_2$]	1.590(4)	1.893(3)	2.097(3)	0.55	
[VO(L^5)$_2$]	1.5988(18)	1.9117(16); 1.9149(16)	2.096(2); 2.098(2)	0.58	9
[VO(L^6)$_2$]	1.601(2)	1.9005(17);	2.076(2); 2.089(2)	0.70	

		1.9027(16)			
$[VO(L^{7})_{2}]$	1.615(8)	1.902(7); 1.900(8)	2.153(9); 2.145(9)	0.43	10
[VO(O⌒N⌒N⌒O)]					
[VO(3-MeOsalen)] $\cdot H_3N(CH_2)_2NH_2Cl$	1.5938(18)	1.9228(16); 1.9287(17)	2.047(2); 2.051(2)	0.13	11
syn-$[VO(HL^{8})_{2}]$	1.596(6)	1.914(4)	2.100(4)	0.55	12
anti-$[VO(HL^{8})_{2}]$	1.577(4)	1.763(3); 1.896(3)	2.064(3); 2.189(3)	0.06	
[VO(salen)]·MeOH	1.6070 (17)	1.9155 (17); 1.9429 (16)	2.051 (2); 2.0597 (19)	0.20	13
$[VOL^{9}]\cdot MeCN$	1.610(5)	1.961(5); 1.972(5)	2.091(6); 2.093(6)	0.03	14
$[VOL^{10}]\cdot CHCl_3$	1.606(2)	1.921(2); 1.922(2)	2.060(3); 2.072(3)	0.29	15
$[VOL^{11}]\cdot CHCl_3$	1.5913(13)	1.9226(12); 1.9351(12)	2.0589(15); 2.0710(15)	0.26	9
$[VOL^{12}]$	1.581(3)	1.937(3); 1.954(3)	2.095(3); 2.100(3)	0.30	
[VO(3-EtOsal-*meso*-stien)]$\cdot H_2O$	1.597(3)	1.925(3); 1.934(2)	2.065(3); 2.072(3)	0.05	16
[VO(sal-*meso*-bn)]	1.595(2)	1.937(2); 1.948(2)	2.061(2); 2.068(2)	0.04	17
$[VO(salen)]ClO_4$	1.576(3)	1.795(3); 1.813(3)	2.074(3), 2.083(3)	0.01	18
[VO{3-EtOsal-(R)-pn}]	1.598(3)	1.918(3); 1.938(3)	2.066(4); 2.053(4)	0.11	19
$[VOL^{13}]$	1.585(3)	1.924(3); 1.938(3)	2.050(3); 2.055(3)	0.12	20
[VO(salen)BF4]	1.577(2)	1.794(2); 1.814(2)	2.067(2); 2.085(2)	0.01	21

HL^{1}	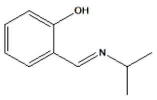	HL^{5}	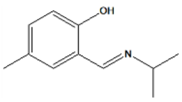	H_2L^{10}	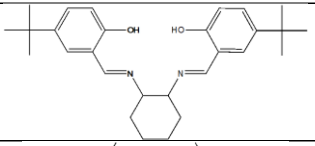
HL^{2}	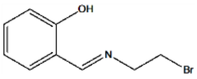	HL^{6}	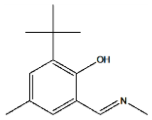	H_2L^{11}	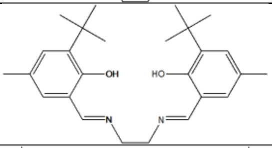
HL^{3}	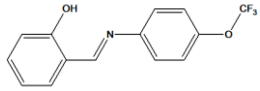	HL^{7}	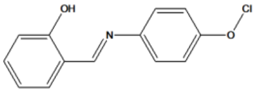	H_2L^{12}	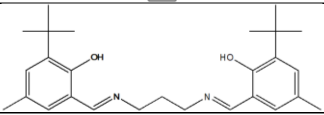
(S)-Clsal-am	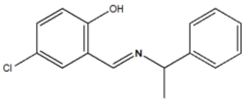	3-MeOsalenH$_2$	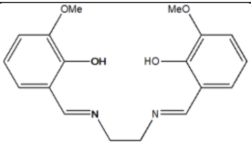	3-EtOsal-*meso*-stienH$_2$	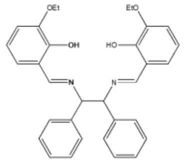
(S)-naph-am	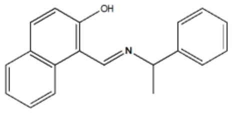	H_2L^{8}	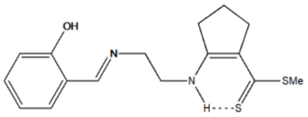	sal-*meso*-bnH$_2$	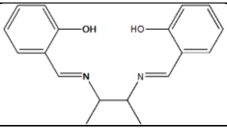
(R)-sal-am	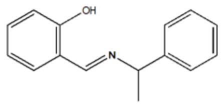	H$_2$salen	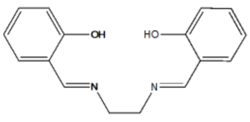	3-EtOsal-(R)-pnH$_2$	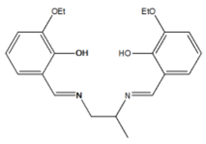

HL^4 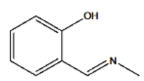	H_2L^9 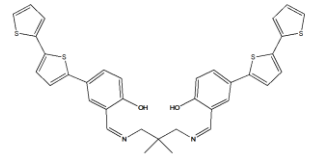	H_2L^{13} 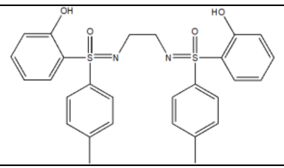

References to table:

1. Shiro M.; Fernando, Q. Structures of two five-coordinated metal chelates of 2-methyl-8-quinolinol. *Anal. Chem.* **1971**, 43, 1222–1230.
2. Groom, C. R.; Bruno, I. J.; Lightfoot M. P.; Ward, S. C. The Cambridge structural database. *Acta Cryst*. **2016**, B72, 171–179
3. Elias, H.; Schwartze-Eidam, S.; Wannowius, K. J. Kinetics and mechanism of ligand substitution in bis(*N*-alkylsalicylaldiminato)oxovanadium(IV) complexes. *Inorg. Chem,* **2003**, 42, 2878–2885.
4. Menati, S.; Rudbari, H. A.; Khorshidifard M.; Jalilian, F. A new oxovanadium(IV) complex containing an O,N-bidentate Schiff base ligand: Synthesis at ambient temperature, characterization, crystal structure and catalytic performance in selective oxidation of sulfides to sulfones using H_2O_2 under solvent-free conditions. *J. Mol. Struct.*, **2016**, 1103, 94–102.
5. Choudhary, N.; Hughes, D. L.; Kleinkes, U.; Larkworthy, L. F.; Leigh, G. J.; Maiwald, M.; Marmion, C. J.; Roger Sanders, J.; Smith G. W.; Sudbrake, C. New tetradentate Schiff bases, their oxovanadium (IV) complexes, and some complexes of bidentate Schiff bases with vanadium (III). *Polyhedron* 1997, **16**, 1517–1528
6. Grivani, G.; Khalaji, A. D.; Tahmasebi, V.; Gotoh K.; Ishida, H. Synthesis, characterization and crystal structures of new bidentate Schiff base ligand and its vanadium(IV) complex: The catalytic activity of vanadyl complex in epoxidation of alkenes. *Polyhedron* **2012**, 31, 265–271.
7. Burgess, J.; Fawcett, J.; Palma V.; Gilani, S. R. Fluoro derivatives of bis(salicylideneaminato-N, O) copper (II) and-oxovanadium (IV). *Acta Cryst.* **2001**, C57, 277–280.
8. Santoni G.; Rehder, D. Structural models for the reduced form of vanadate-dependent peroxidases: vanadyl complexes with bidentate chiral Schiff base ligands. *J. Inorg. Biochem.* **2004**, 98, 758–764.
9. Cornman, C. R.; Geiser-Bush, K. M.; Rowley, S. P.; Boyle, P. D. Structural and Electron Paramagnetic Resonance Studies of the Square Pyramidal to Trigonal Bipyramidal Distortion of Vanadyl Complexes Containing Sterically Crowded Schiff Base Ligands. *Inorg. Chem.* **1997**, 36, 6401–6408.
10. Pasquali, M.; Marchetti, F.; Floriani C.; Merlino, S. Oxovanadium(IV) complexes containing bidentate Schiff-base ligands: synthesis and structural and spectroscopic data. *J. Chem. Soc., Dalton Trans.* **1977**, 139–144.
11. Cashin, B.; Cunningham, D.; Daly, P.; McArdle, P.; Munroe M.; Chonchubhair, N. N. Donor properties of the vanadyl ion: Reactions of vanadyl salicylaldimine β-ketimine and acetylacetonato complexes with groups 14 and 15 Lewis acids. *Inorg. Chem.* **2002**, 41, 773–782.
12. Bhattacharyya, S.; Mukhopadhyay, S.; Samanta, S.; Weakley, T. J. R.; Chaudhury, M.; Synthesis, characterization, and reactivity of mononuclear O,N-chelated vanadium(IV) and -(III) complexes of methyl 2-aminocyclopent-1-ene-1-dithiocarboxylate based ligand: Reporting an example of conformational isomerism in the solid state. *Inorg. Chem.* **2002**, 41, 2433–2440
13. Hsuan, R. E.; Hughes, J. E.; Miller, T. H.; Shaikh, N.; Cunningham, P. H. M.; O'Connor, A. E.; Tidey J. P.; Blake, A. J. Crystal structure of {2,2'-[ethylenebis(nitrilomethanylylidene)]diphenolato-κ^4*O*,*N*,*N*',*O*'}oxidovanadium(IV) methanol monosolvate. *Acta Cryst*. **2014**, E70, m380–m381.
14. Nguyen, M. T.; Jones R. A.; Holliday, B. J. Effect of conjugation length and metal-backbone interactions on charge transport properties of conducting metallopolymers. *Polym. Chem.* **2017**, 8, 4359–4367.
15. Carter, E.; Fallis, I. A.; Kariuki, B. M.; Morgan, I. R.; Murphy D. M.; Tatchell, T.; Van Doorslaer S.; Vinck, E. Structure and pulsed EPR characterization of *N*,*N*′-bis(5-*tert*-butylsalicylidene)-1,2-cyclohexanediamino-vanadium(IV) oxide and its adducts with propylene oxide. *Dalton Trans.*, **2011**, 40, 7454–7462.

16. Hoshina, G.; Tsuchimoto, M.; Ohba, S.; Nakajima, K.; Uekusa, H.; Ohashi, Y.; Ishida H.; Kojima, M. Thermal dehydrogenation of oxovanadium(IV) complexes with schiff base ligands derived from *meso*-1,2-diphenyl-1,2-ethanediamine in the solid state. *Inorg. Chem.* **1998**, 37, 142–145.
17. Hoshina, G.; Tsuchimoto M.; Ohba, S. *exo*-[(*RS*,*SR*)-*N*,*N*'-Bis(salicylidene)-2,3-butanediaminato]oxovanadium(IV) *Acta Cryst.* **1999**, C55, 1082–1084.
18. Bonadies, J. A.; Butler, W. M.; Pecoraro V. L.; Carrano, C. J. Novel reactivity patterns of (N, N'-ethylenebis (salicylideneaminato)) oxovanadium (IV) in strongly acidic media. *Inorg. Chem.* **1987**, 26, 1218–1222.
19. Hoshina, G.; Tsuchimoto M.; Ohba, S. *endo*-{6,6'-Diethoxy-2,2'-[(*R*)-propane-1,2-diylbis(nitrilomethylidene)]diphenolato-*O*,*N*,*N*',*O*'}oxovanadium(IV), *Acta Cryst.* **1999**, C55, 1812-1813.
20. Bolm, C.; Bienewald F.; Harms, K. Syntheses and vanadium complex of salen-like bissulfoximines, *Synlett*, 1996, 8, 775–776.
21. Oyaizu, K.; Dewi, E. L.; Tsuchida, E. Coordination of BF_4^- to Oxovanadium(V) Complexes, Evidenced by the Redox Potential of Oxovanadium(IV/V) Couples in CH_2Cl_2. *Inorg. Chem.* **2003**, 42, 1070–1075.

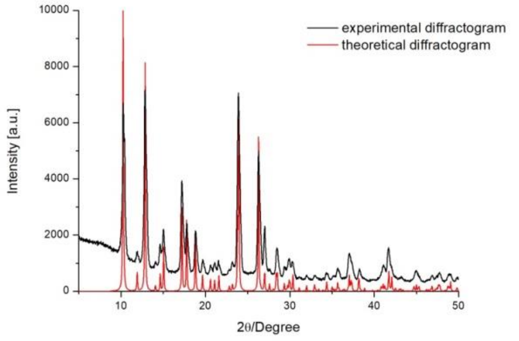

1

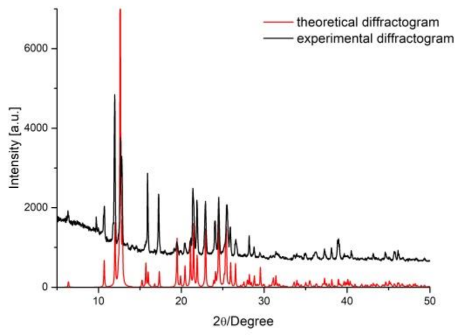

2

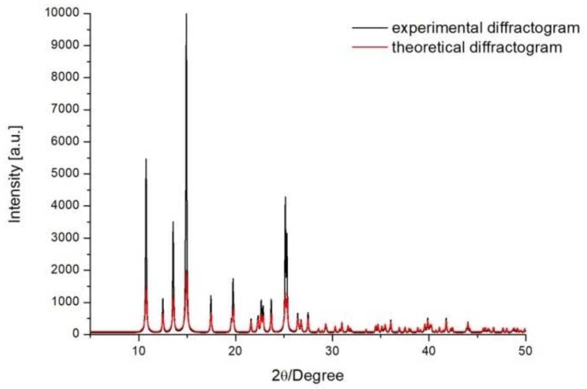

3

Figure S9. X–ray powder diffraction pattern of **1–3** together with the calculated pattern from the single crystal data.

EPR spectra

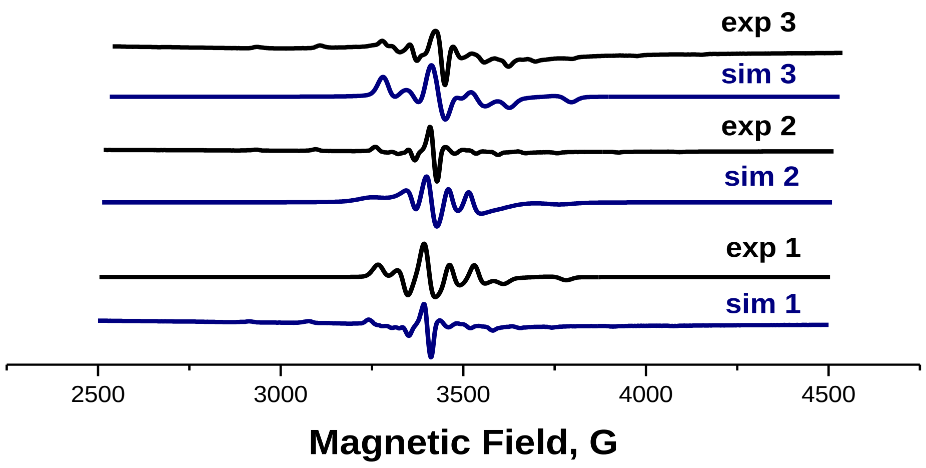

Figure S10. EPR frozen solution spectra (at 77 K) of compounds **1–3**; in acetonitrile solvent together with the theoretical spectrum calculated using the parameters: $g_x = g_y = 1.980$, $g_z = 1.942$, $A_x = A_y = 65$ G, $A_z = 181$ G for **1** and $g_x = g_y = 1.979$, $g_z = 1.957$, $A_x = A_y = 53$ G, $A_z = 169$ G for **2;** $g_x = g_y = 1.978$, $g_z = 1.959$, $A_x = A_y = 55$ G, $A_z = 168$ G for **3**

Absorption spectroscopy

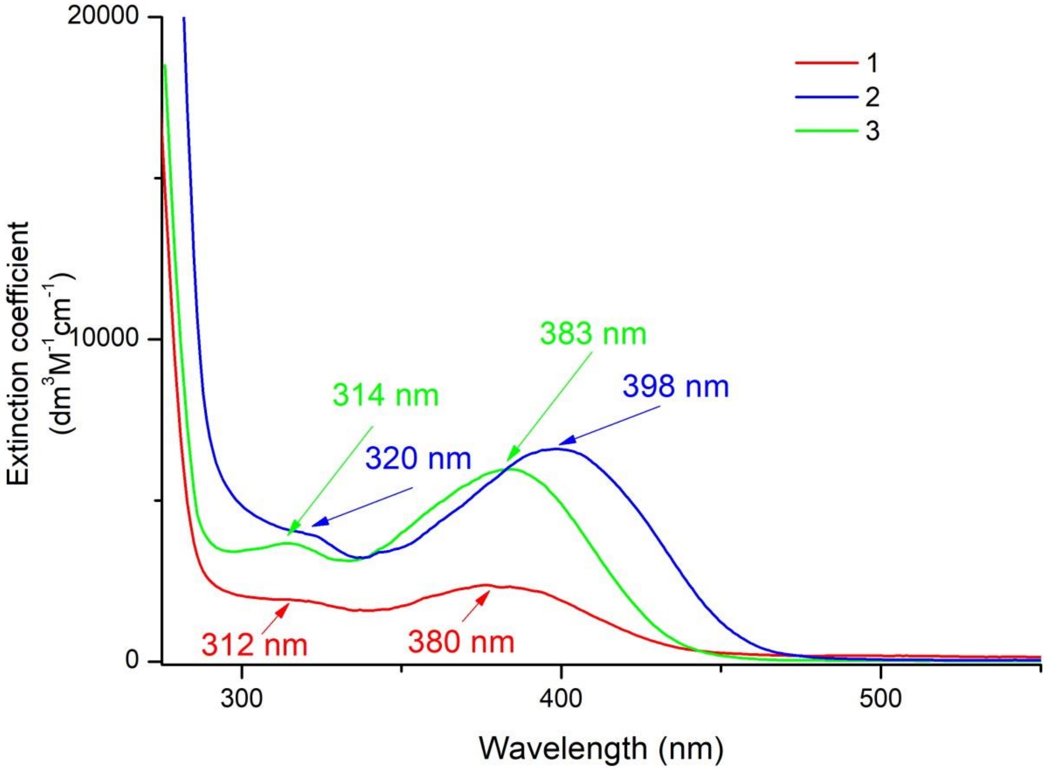

(a)

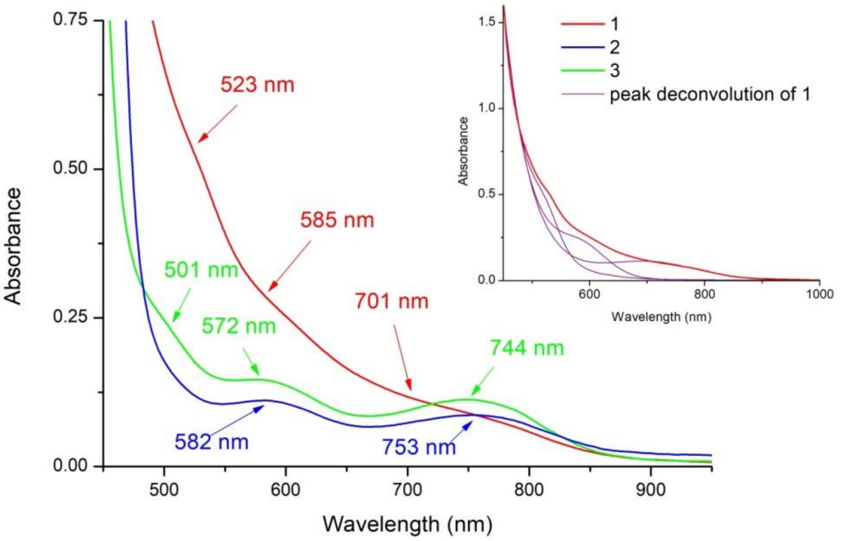

(b)

Figure S11. UV-Vis spectra of **1–3** in DMSO full scale ($5{\cdot}10^{-5}$ M) (a), close-up of the 500–900 nm region (concentrated solutions in DMSO) (b), insert: peak deconvolution of **1** calculated using deconvolution formula implemented in the OriginPro 9.1 software

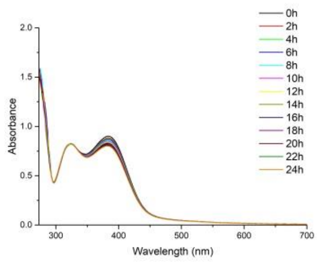	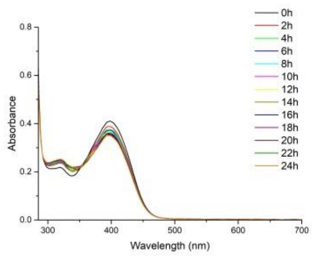	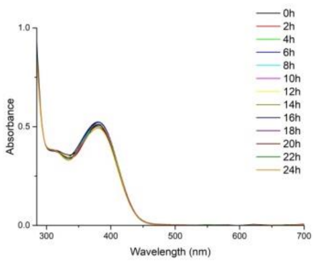
**(a)**	**(b)**	**(c)**
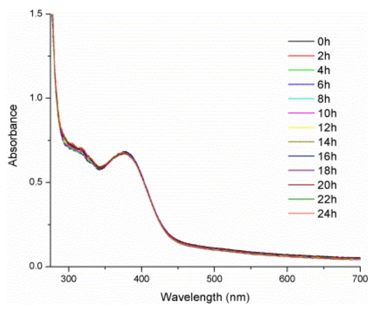	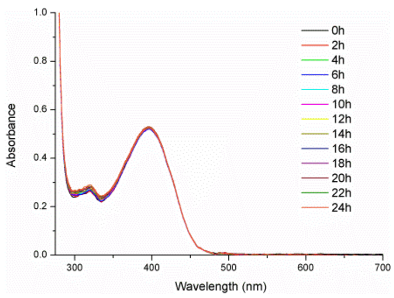	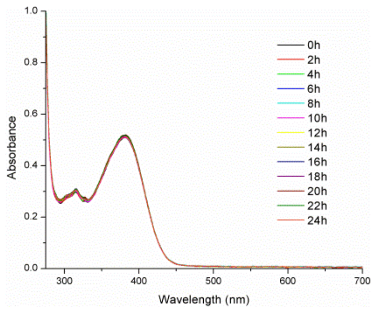
**(d)**	**(e)**	**(f)**

Figure S12. UV-Vis stability spectra for compounds **1–3** in DMSO (10^{-4} M) (a-c) and CH_3CN (d-f). Spectra were recorded every 2 hours for 24h.

Catalytic data

Table S8. Comparative parameters for the oxidation of cyclohexane catalyzed by complexes 1-3 and other vanadium complexes previously published

Catalytic system	Conditions	Vanadium oxidation state	Total yield of oxygenates (%)	Total TON	Ref.
[n-Bu$_4$N][VO$_3$]/pca/H$_2$O$_2$/CH$_3$CN	40 °C, 4 h	V	30	700	1
[n-Bu$_4$N][VO$_2$(pca)$_2$]/H$_2$O$_2$/CH$_3$CN	40 °C, 24 h	V	4.6	215	2
[n-Bu$_4$N][VO$_2$(pca)$_2$]/pca/H$_2$O$_2$/CH$_3$CN	40 °C, 24 h	V	20	900	
[VO(OCH$_3$)(ma)2]<br>Hma – 3-hedroxy-2-methyl-4-pyrone	40°C, 14 h	V	27	2600	3
[VO(tea)]<br>tea – triethanolamine	40 °C, 24 h	V	10	900	4
[VO(OCH3)(5-Cl-quin)$_2$ ·1/2CHCl3/pca/H$_2$O$_2$/CH$_3$CN<br>5-Cl-quin – 5-chloro-8-hydroxyquinoline	40°C, 6 h	V	39	360	5
[{VO(Oet)(EtOH)}$_2$L]/pca/H$_2$O$_2$/CH$_3$CN<br>H$_4$L – bis(2-hydroxybenzylidene)terephthalohydrazide	50 °C, 24 h	V	30	700	6
[{VO(Oet)(EtOH)}$_2$L]/pca/H$_2$O$_2$/CH$_3$CN<br>H$_4$L – bis(2-hydroxybenzylidene)oxalohydrazonic acid	50 °C, 4 h	V	28.7	2640	7
[VO$_2$(pca)(hmpa)]/pca/H$_2$O$_2$/CH$_3$CN<br>hmpa – hexamethylphosphoramide	40 °C, 24 h	V	29.8	1370	8
[VO$_2$(pycaH)(hmpa)]/pca/H$_2$O$_2$/CH$_3$CN<br>hmpa – hexamethylphosphoramide<br>pycaH$_2$ – pyridine-2,5-dicarboxylic acid	40 °C, 24 h	V	24.8	1140	
[(VO$_2$)$_2$(μ$_4$-L){Na$_2$(μ-H$_2$O)$_2$(H$_2$O)$_2$}]$_n$/pca/H$_2$O$_2$/CH$_3$CN<br>H$_4$L – bis(salicylaldehyde)-oxaloyldihydrazone	50 °C, 5 h	V	4.8	4400	9
[{V(μ-O)$_2$}$_2$(μ$_4$-L){K$_2$(μ-H$_2$O)$_2$(H$_2$O)$_2$}]$_n$/pca/H$_2$O$_2$/CH$_3$CN<br>H$_4$L – bis(salicylaldehyde)-oxaloyldihydrazone	50 °C, 5 h	V	5.7	5220	
[{V(μ-O)(μ$_3$-O)}2(μ$_8$-L){Cs$_2$(μ-H$_2$O)$_2$(H$_2$O)$_2$}]$_n$<br>/pca/H$_2$O$_2$/CH$_3$CN<br>H$_4$L – bis(salicylaldehyde)-oxaloyldihydrazone	50 °C, 5 h	V	6.2	5700	
V$_3$O$_3$(Oet)(ashz)$_2$(μ-Oet)]$_2$/pca/H$_2$O$_2$/CH$_3$CN<br>H$_3$ashz – N-acetylsalicylhydrazidate	50 °C, 5 h	mixed IV/V	18.2	8370	10
[(VO)$_4$(hptb)$_2$(H$_2$O)$_2$(μ-O)][ClO$_4$]/pca/H$_2$O$_2$/CH$_3$CN<br>hptbH – N,N,N′,N′-tetrakis(2-benzimidazolylmethyl)-2-hydroxo-1,3-diaminopropane	40 °C, 24 h	IV	12.4	570	8
[VOCl$_2$(tmtacn)]/pca/H$_2$O$_2$/CH$_3$CN<br>tmtacn – 1,4,7-trimethyl-1,4,7-triazacyclononane	40 °C, 24 h	IV	6.3	290	
[V(cat)$_3$]$^-$/pca/H$_2$O$_2$/CH$_3$CN<br>catH$_2$ – pyro-cathecol	40 °C, 24 h	IV	21.3	980	
[VOCl$_2$(dpp-bian)]/pca/H$_2$O$_2$/ CH$_3$CN<br>dpp-bian – bis(N-(2,6-diisopropylphenyl)-imino)acenaphthene	50 °C, 5 h	IV	21.5	990	11
VO(acac)$_2$/H$_2$O$_2$/ CH$_3$CN	40°C, 5 h	IV	1.2	23	12,13
1/pca/ H$_2$O$_2$/CH$_3$CN	50 °C, 3 h	IV	30	565	this work
2/pca/ H$_2$O$_2$/CH$_3$CN	50 °C, 2 h	IV	43	870	
3/pca/ H$_2$O$_2$/CH$_3$CN	50 °C, 1.5 h	IV	48	954	

References to table:

1. Shul'pin, G. B.; Attanasio, D. ; Suber, L. Efficient H_2O_2 oxidation of alkanes and arenes to alkyl peroxides and phenols catalyzed by the system vanadate–pyrazine-2-carboxylic acid. *J. Catal*. **1993**, 142, 147–152.
2. Süss-Fink, G.; Stanislas, S.; Shul'pin, G. B.; Nizova, G. V.; Stoeckli-Evans, H.; Neels, A.; Bobillier, C.; Claude, S.; Oxidative functionalisation of alkanes: synthesis, molecular structure and catalytic implications of anionic vanadium(V) oxo and peroxo complexes containing bidentate N,O ligands. *J. Chem. Soc., Dalton Trans.* **1999**, 3169-3175.
3. Shul'pin, G. B.; Mishra, G. S.; Shul'pina, L. S.; Strelkova, T. V.; Pombeiro, A. J. L. Oxidation of hydrocarbons with hydrogen peroxide catalyzed by maltolato vanadium complexes covalently bonded to silica gel. *Catal. Commun*. **2007**, 8, 1516–1520.
4. Kirillova, V.; Kuznetsov, M. L.; Romakh, V. B.; Shul'pina, L. S.; Fraústo da Silva, J. J. R.; Pombeiro, A. J. L.; Shul'pin, G. B. Mechanism of oxidations with H_2O_2 catalyzed by vanadate anion or oxovanadium(V) triethanolaminate (vanadatrane) in combination with pyrazine-2-carboxylic acid (PCA): Kinetic and DFT studies. *J. Catal*. **2009**, 267, 140–157.
5. Sutradhar, M.; Shvydkiy, N. V.; da Silva, M. F. C. G.; Kirillova, M. V.; Kozlov, Y. N. ; Pombeiro, A. J. L.; Shul'pin, G. B. New binuclear oxovanadium(V) complex as a catalyst in combination with pyrazinecarboxylic acid (PCA) for efficient alkane oxygenation by H_2O_2, *Dalton Trans*., **2013**, 42, 11791–11803.
6. Sutradhar, M.; Shvydkiy, N. V.; Guedes da Silva, C. F. M.; Kirillova, M. V.; Kozlov, Y. N.; Pombeiro, A. J. L.; Shul'pin, G. B. A new binuclear oxovanadium(v) complex as a catalyst in combination with pyrazinecarboxylic acid (PCA) for efficient alkane oxygenation by H2O2. *Dalton Trans*., **2013**, 42, 11791-11803.
7. Gupta, S.; Kirillova, M. V.; Guedes da Silva, M. F.; Pombeiro, A. J. L., Highly efficient divanadium(V) pre-catalyst for mild oxidation of liquid and gaseous alkanes. *Appl. Catal. A-Gen.,* **2013**, 460-461, 82–89.
8. Süss-Fink, G.; Gonzalez Cuervo, L.; Therrien, B.; Stoeckli-Evans, H.; Shul'pin, G. B. Mono and oligonuclear vanadium complexes as catalysts for alkane oxidation: synthesis, molecular structure, and catalytic potential. *Inorg. Chim. Acta,* **2004**, 357, 475–484.
9. Gupta, S.; Kirillova, M. V.; Guedes da Silva, M. F. C.; Pombeiro, A. J. L.; Kirillov, A. M. Alkali metal directed of heterometallic V^{v}/M (M = Na, K, Cs) coordination polymers: structures, topological analysis, and oxidation catalytic properties. *Inorg. Chem*. 2013, 52, 15, 8601–8611.
10. Sutradhar, M.; Kirillova, M. V.; Guedes da Silva, M. F. C.; Martins, L. M. D. R. S; Pombeiro, A. J. L. A hexanuclear mixed-valence oxovanadium(IV,V) complex as a highly efficient alkane oxidation catalyst. Inorg. Chem. **2012**, 51, 11229–11231.
11. Fomenko, I. S.; Gushchin, A. L.; Shul'pina, L. S; Ikonnikov, N. S.; Abramov, P. A.; Romashev, N. F.; Poryvaev, A. S.; Sheveleva, A. M; Bogomyakov, A. S.; Shmelev, N. Y.; Fedin, M. V.; Shul'pin, G. B.; Sokolov, M. N. New oxidovanadium(iv) complex with a BIAN ligand: synthesis, structure, redox properties and catalytic activity. New J. Chem., **2018**, 42, 16200-16210.
12. Pokutsa, A.; Bloniarz, P.; Fliunt, O.; Kubaj, Y.; Zaborovskyi, A.; Pacześniak, T. Sustainable oxidation of cyclohexane catalyzed by a VO(acac)2-oxalic acid tandem: the electrochemical motive of the process efficiency. *RSC Adv.* **2020**, 10, 10959–10971.
13. Pokutsa, A.; Kubaj, Y.; Zaborovskyi, A.; Sobkowiak, A., Muzart, J. Oxalic acid-improved mild cyclohexane oxidation catalyzed by VO(acac)2: non-radical versus radical mechanism. *Reac. Kinet. Mech. Cat.* **2017,** 122, 757–774.

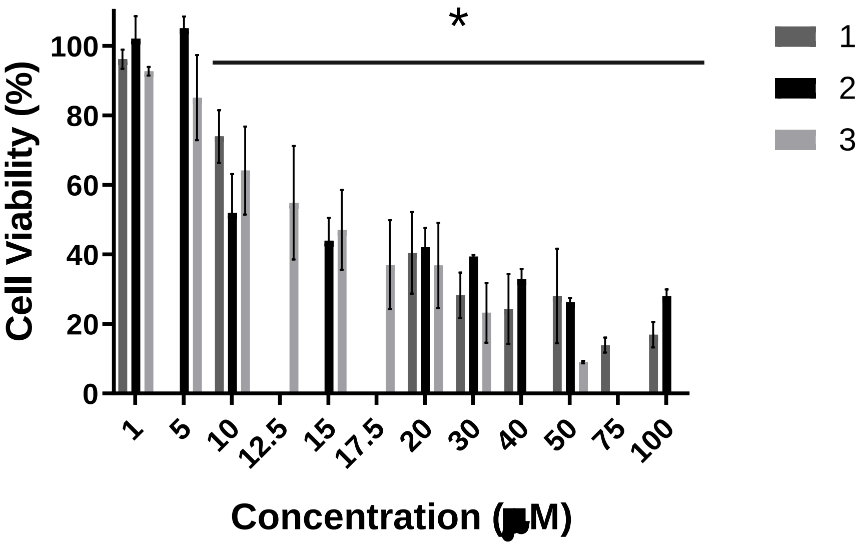

Figure S13. Antiproliferative effect of complexes **1-3** in normal dermal fibroblasts, after 48 h. evaluated by the MTS method. Cell viabilities were normalized to DMSO 0.1% (v/v) (vehicle control). The results presented are mean ± standard deviation of three independent assays. Asterisk indicates a p-values inferior to 0.05

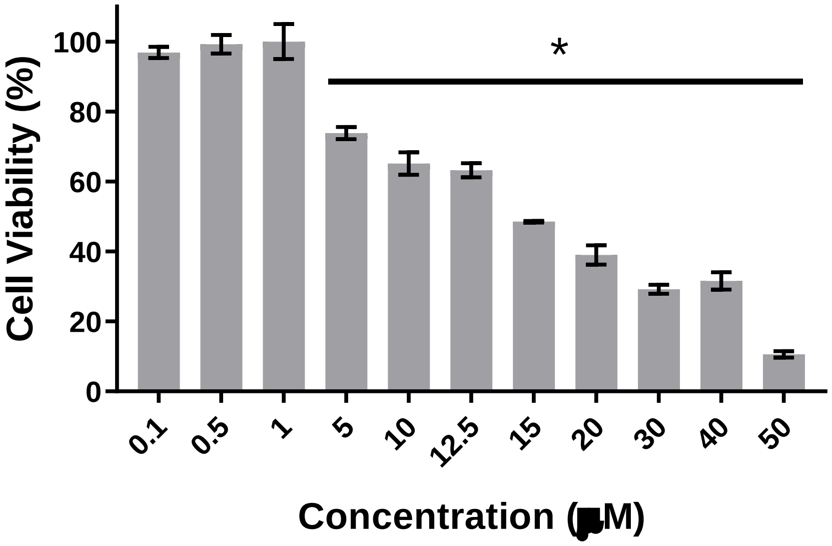

Figure S14. Cell viability of HCT116 cells exposed for 48 h to increasing concentrations of cisplatin. IC_{50} value found is 15.6 ± 5.3 μM.`* p<0.05 relative to control.
